# GOAT – A simple LC-MS/MS gradient optimization tool

**DOI:** 10.1002/pmic.201300524

**Published:** 2014-05-15

**Authors:** David C Trudgian, Roman Fischer, Xiaofeng Guo, Benedikt M Kessler, Hamid Mirzaei

**Affiliations:** 1 Department of Biochemistry, University of Texas Southwestern Medical CenterDallas, TX, USA; 2 Target Discovery Institute, Nuffield Department of Medicine, University of OxfordOxford, UK

**Keywords:** Bioinformatics, Gradient, LC-MS/MS, Liquid chromatography, Optimization, Separation

## Abstract

Modern nano-HPLC systems are capable of extremely precise control of solvent gradients, allowing high-resolution separation of peptides. Most proteomics laboratories use a simple linear analytical gradient for nano-LC-MS/MS experiments, though recent evidence indicates that optimized non-linear gradients result in increased peptide and protein identifications from cell lysates. In concurrent work, we examined non-linear gradients for the analysis of samples fractionated at the peptide level, where the distribution of peptide retention times often varies by fraction. We hypothesized that greater coverage of these samples could be achieved using per-fraction optimized gradients. We demonstrate that the optimized gradients improve the distribution of peptides throughout the analysis. Using previous generation MS instrumentation, a considerable gain in peptide and protein identifications can be realized. With current MS platforms that have faster electronics and achieve shorter duty cycle, the improvement in identifications is smaller. Our gradient optimization method has been implemented in a simple graphical tool (GOAT) that is MS-vendor independent, does not require peptide ID input, and is freely available for non-commercial use at http://proteomics.swmed.edu/goat

The introduction of non-split flow nano-HPLC systems to LC-MS/MS proteomics workflows has allowed improved coverage of samples via high-resolution separation of peptides. Modern nano-HPLC systems are capable of accurately delivering extended solvent gradients at low-flow rates and high pressures through long capillary columns packed with low pore-size resins 1. These capabilities increase the peak capacity of the online reversed-phase (RP) LC separation, simplifying the mixture of peptides delivered to the mass-spectrometer at a point in time during an LC-MS/MS analysis and allowing deeper coverage of the sample 2. The accuracy of solvent delivery for very shallow gradients in the nanoliter per minute flow range using modern binary pumps allows multi-hour LC-MS/MS analyses to be performed successfully. Various studies have made use of gradients up to 6 h in length to identify thousands of proteins from unfractionated lysates 3–5.

Despite the improvements in coverage of unfractionated samples resulting from LC and MS advances, fractionation prior to MS analysis is required for deepest coverage. This pre-fractionation is commonly performed at the peptide-level, after the sample has been digested. Increased coverage is due to the fact that the pre-fractionation method employed is orthogonal to the RP separation in the LC-MS/MS analysis. Techniques commonly applied include column-based methods such as strong-anion exchange (SAX), strong-cation exchange, and high-pH RP 6, as well as IEF 7. The resulting fractions are subjected to LC-MS/MS typically in the same manner as unfractionated samples–using a simple linear gradient of ACN, e.g. 2–35% over 1–4 h followed by a fast ramp to a high organic concentration, e.g. 35–80% ACN in 5 min. The use of this simple gradient for all fractions neglects the fact that the pre-fractionation methods are not truly orthogonal to the online RPLC and can introduce a bias toward low or high hydrophobicity between fractions 8.

If a peptide fraction predominantly contains either hydrophilic or hydrophobic peptides, the online RP gradient is not used efficiently. Figure1 shows total ion current (TIC) chromatograms for a high-pH RP fractionation of *Jurkat* cell lysate analyzed using a simple linear gradient. Flow-through and three elutions at 10, 20, and 50% ACN are shown. It is clear that peptide hydrophobicity varies between fractions, and a different portion of the gradient is under-used in each sample. This effect is observed frequently by proteomics laboratories such as our own. However, the standard simple linear gradient is generally applied to the analysis of pre-fractionated samples.

**Figure 1:**
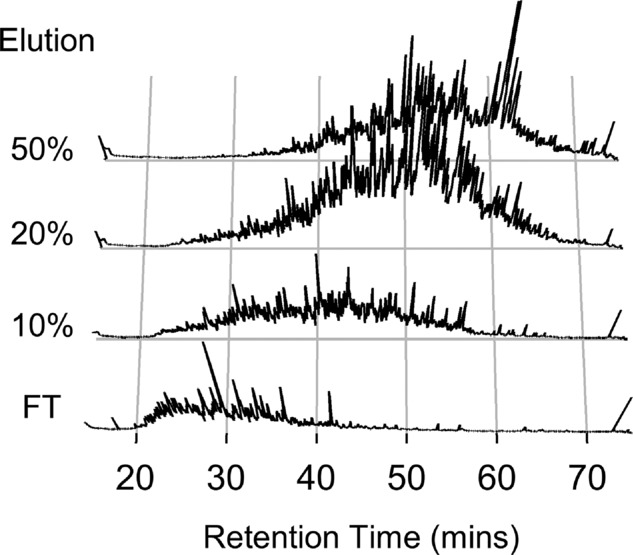
Hydrophobicity bias in peptide-level fractionation. A four-fraction high pH reverse phase separation of *Jurkat* cell lysate digest was analyzed using standard 60-min analytical gradient LC-MS/MS methods. Total ion current chromatograms for each fraction clearly show differences in the retention time distribution of peptides in these fractions.

Recently, Moruz et al. 9 demonstrated the utility of generating optimized non-linear gradients for LC-MS/MS analyses. The authors showed that the retention times of peptides in unfractionated *HeLa* lysate were not distributed evenly in a simple 2–32% gradient of ACN. Using optimized non-linear gradients, improvements in peptide identifications of up to 10% were demonstrated. In concurrent work, we observed a 5% increase in identifications from unfractionated whole cell lysate during initial investigation of gradient optimization (see Supporting Information). We hypothesized that optimized gradients would have greater benefits for samples pre-fractionated at the peptide level, due to the differing distribution of peptide hydrophobicity between fractions. We also aimed to provide a simple graphical tool for gradient optimization that is MS vendor independent, and does not require the user to be comfortable with command line scripts.

To examine the benefits of gradient optimization for fractionated samples, tryptic digests of whole cell lysates were prepared at two institutions using methods commonly employed by each. In the first laboratory, at the University of Oxford, high-pH RP separation was performed (hpRP) to generate four fractions from *Jurkat* lysate digests comprising flow-through and elutions using 10, 20, and 50% ACN in ammonium formate at pH10. Conditions were chosen with reference to Gilar et al. 10. LC-MS/MS analysis was performed using a nanoAcquity UPLC system (Waters, Milford, MA, USA), coupled to an LTQ-Orbitrap Velos or a U3000-RLSCnano system (Dionex, Sunnyvale, CA, USA) on a Q Exactive mass-spectrometer (Thermo Fisher, Bremen). Data were acquired using a standard analytical gradient of 0–40% ACN in 60 or 120 min (see Supporting Information Methods for details). In the second laboratory, at UT Southwestern Medical Center, a separate *HeLa* digest was fractionated using a tip-based SAX protocol into eight fractions with elution at pH 2, 3, 4, 6, 8, and 11, and finally 80% ACN. LC-MS/MS analysis was performed using a U3000-RLSCnano system coupled to an Orbitrap Elite MS (Thermo Fisher, Bremen). The standard analytical gradient was 2–25% ACN in 100 min. Full details of fractionation and MS are given in the Supporting Information Methods. The diversity of pre-fractionation methods and mass-spectrometers covers some of the most common methods and platforms used in proteomics facilities, demonstrating that GOAT is useful for a variety of laboratories and workflows.

Based on data acquired from the standard gradients, optimized gradients were computed using our GOAT tool. Our intention was to create a tool that did not require MS1 peak-picking or peptide-identification input to drive gradient optimization. MS1 peak-picking can accurately identify peptide precursor ions visible in a sample, whether or not they have been identified. However, peak-picking algorithms are computationally complex and must be optimized for the characteristics of different MS instruments 11. Peptide identification information for gradient optimization can be obtained by searching data acquired on a standard gradient. However, peptide ID results are produced by different search engines in a variety of formats, requiring multiple import filters or manual manipulation to use in a generic optimization tool. Additionally, many workflows do not report retention times for peptides in their output. In silico gradient optimization, as performed by Moruz et al. would be complex for fractionated or enriched samples, as the set of peptides used for per-fraction optimization must accurately reflect the separation or enrichment employed.

To obtain a simple set of points for optimization, we make the assumption that a certain percentage of spectra in an LC-MS/MS run can be identified *(i)*, and that these identifiable MS/MS spectra have the highest TICs in the sample. To create an optimized gradient, we extract the retention time for all MS/MS scans and then filter this set to the highest *i*% by TIC. The MS retention time is mapped to a% solvent B value using the user-provided standard gradient, LC dead-volume delay, and an optional MS start delay. The result is a list of *N* MS/MS spectra and the% solvent B at which they eluted. A non-linear gradient is constructed from *S* linear steps, at intervals of *T* minutes. The% solvent B at each step is chosen so that all steps contain an equal fraction of the *N* MS/MS spectra acquired in the standard gradient. This MS/MS scan based optimization allows gradient optimization to be performed extremely quickly, without the need for peak-picking optimization or peptide IDs.

To create an optimized gradient with our GOAT tool, the user must provide a raw format or MzXML/MzML data file generated using a standard gradient, details of that gradient, and the LC dead volume delay. Note that Moruz et al. provided a simple procedure to obtain an accurate value for the LC dead volume delay. Three optimization parameters can be adjusted, with sensible defaults (used in this study) provided. MS/MS scans with precursor charge states below a specified charge can be excluded from the optimization. By default, we exclude singly charged precursors as many are chemical contaminants. The user may also specify a value for *i –* the percentage of MS/MS spectra typically identified. Here we used 50%, which is a typical value for complex lysate fractions on Orbitrap and Q Exactive instruments in our experience. The resolution of the optimization *T* can also be altered and is 5 min by default. The optimization procedure is illustrated in Fig.2A, with the standard and optimized gradient shown in Fig.2B for the pH3 elution of the SAX separation. Our software uses the Proteowizard MSaccess utility 12 to extract scan information from raw files in a vendor-independent manner.

**Figure 2:**
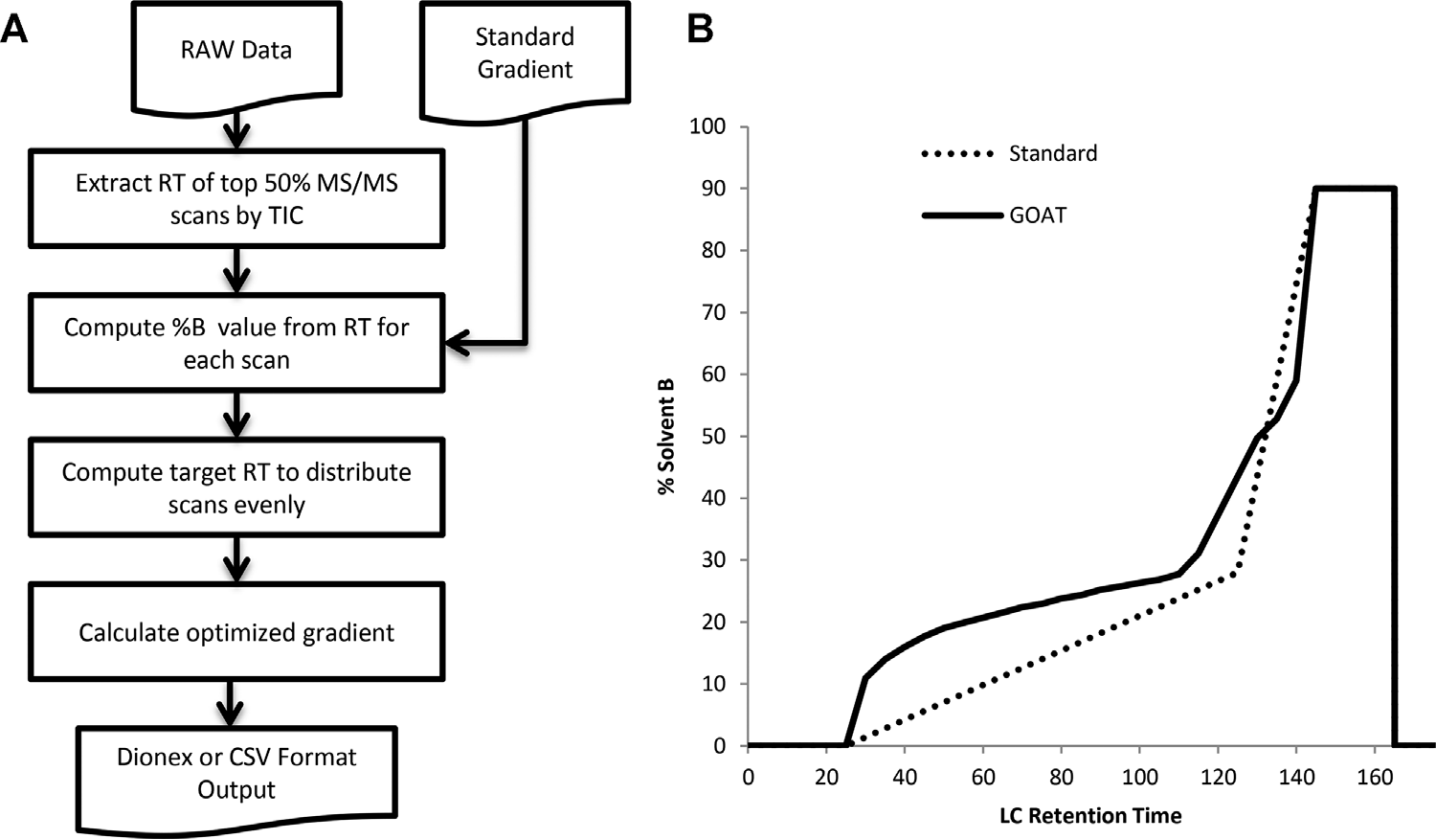
GOAT gradient optimization process and output. (A) Flowchart showing the procedure used to optimize LC gradient in our GOAT tool. (B) Example standard and optimized gradients for the pH 3 elution of the SAX fractionated HeLa lysate.

To assess the improvement offered by gradient optimization, we analyzed each dataset using our in-house central proteomics facilities pipeline 13. Briefly, all data were searched against the UniProtKB whole proteome sequence database (release 2012_04), considering up to three missed cleavages, and oxidation of Met as a variable modification. Ion tolerances were 20 ppm for precursors and 0.1 Da or 0.5 Da for fragments on Q Exactive and Orbitrap data, respectively. Peptide to spectrum match, unique peptide sequence, and protein group counts were obtained at a 1% FDR using the concatenated target-decoy method 14. Full details of data processing, protein identifications, and gradients are given for each dataset in the Supporting Information Methods and Supporting Information Tables 1-15.

GOAT optimization of gradients improves the distribution of peptides throughout the length of the analysis, as seen in Fig.3A, which displays base-peak chromatograms for the pH 3 SAX elution. In the standard gradient, the majority of material elutes close to the end of the run, while the GOAT gradient makes use of a far greater portion of the analysis. Figure3B plots the increase in peptide to spectrum matches (total spectral counts), and protein groups for each dataset. We observed the greatest increase in identifications for short 1-h gradients on previous generation Orbitrap Velos instrumentation, and the smallest improvement for data acquired using a current Q Exactive instrument. The magnitude of the improvement correlates with the instrument duty cycle (Supporting Information Fig. 1). The slower Orbitrap Velos undersamples on the standard gradient, and identifications increase when peptides are distributed more evenly through the runs. The Q Exactive instrument is sampling sufficiently fast that there is little improvement in IDs. The Orbitrap Elite has a slightly longer duty cycle than the Q Exactive, and shows some increase in peptide and protein ID. These findings are in agreement with Moruz et al. who observed greater improvements in unfractionated lysate for 2-h gradients on slower LTQ-Orbitrap XL instrumentation than 4-h gradients using a Q Exactive.

**Figure 3:**
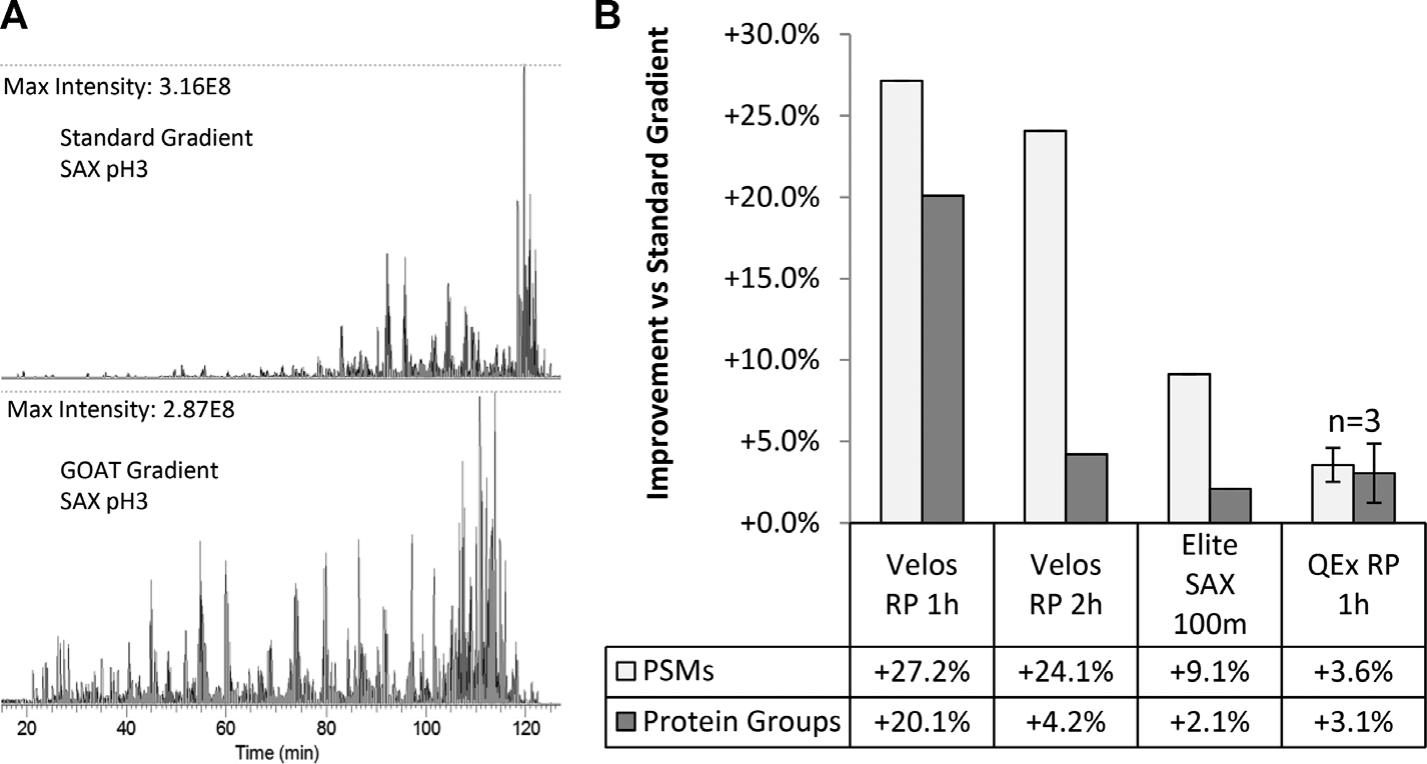
Improvement in peptide and protein identification achieved using optimized gradients. (A) GOAT optimization dramatically spreads the elution of peptides in a pH 3 SAX elution, demonstrated with base peak chromatograms. (B) A summary of the increase in identifications observed on SAX and high-pH RPLC fractionated samples, at two institutions on three different instrumentation platforms. Slower instrumentation exhibits the greatest identification improvement with optimized gradients. Q Exactive RP values are mean improvements across three replicates, with SD shown as error bars. Other values are from single injections of each set of fractions.

Qualitatively, our results suggest that optimized gradients are beneficial for the analysis of peptide-fractionated samples, particularly on slower duty cycle mass spectrometers. We have demonstrated that by using only MS/MS retention time and TIC information, we can successfully increase peptide and protein identifications. Though it is not practical to run standard and optimized gradients for all samples, GOAT may be used as part of the development of a standardized pre-fractionation method. Working from a standard protein mixture, optimized gradients can be developed for each fraction, which can then be re-used on similar experimental samples. Laboratories may use GOAT to assemble a library of gradients for each pre-fractionation approach employed, and use these gradients without the need to perform optimization on each sample. On slower instrumentation, such as the Orbitrap Velos and earlier, significant gains in peptide and protein identification can be achieved. Despite the low ID improvement on more recent faster instrumentation, we believe the method would be applicable to experiments where enrichment methods deliver samples that are heavily biased toward hydrophobic or hydrophillic peptides. There are also applications in targeted proteomics: in complex scheduled SRM studies, which assay a large number of peptides, elution must be uniform throughout the run to minimize duty cycle. Gradient optimization from shotgun data may be useful for this task. Outside of proteomics, GOAT can be used to optimize the separation of any mixture of molecules studied in a data-dependent LC-MS/MS experiment. Since gradient optimization is based on acquired MS/MS scan timings and TIC, it is not specific to peptides, and could be applied to metabolites, lipids, etc.

Our GOAT software is simple to use. It is a graphical application for Windows XP and above, which requires the .NET framework (v4) and an installation of the ProteoWizard tools with vendor support. Because optimization is performed using MS/MS scan information, our tool is instrument and vendor independent. The simplicity of the procedure allows gradients to be generated quickly from large RAW files. All parameters are adjusted in the application via simple controls. Gradients are visualized within GOAT and can be copied to the clipboard for transfer into LC control software. Tab-separated and Dionex Chromeleon command formats are supported. Download at: http://proteomics.swmed.edu/goat/

## Abbreviations

SAX: strong-anion exchange

## Supporting Information

As a service to our authors and readers, this journal provides supporting information supplied by the authors. Such materials are peer reviewed and may be re-organized for online delivery, but are not copy-edited or typeset. Technical support issues arising from supporting information (other than missing files) should be addressed to the authors.

**Figure S1 –** Improvement in number of peptide-spectrum matches from GOAT gradient optimization correlates inversely with the speed of the instrument used to acquire the data.

Tables S1 – S10 list protein identifications for all datasets discussed in the main text. Data were acquired at two sites, using 3 different instruments and a variety of standard and GOAT optimized gradients. See Supporting Information Methods for details.

Tables S11 – S15 list standard and GOAT optimized gradients for each experiment.
