# Supplementary material for: GOAT – A simple LC-MS/MS gradient optimization tool
Source: Proteomics. 2014 May 15;14(12):1467–71. doi: 10.1002/pmic.201300524 (PMC4375517; doi:10.1002/pmic.201300524)
Supplement: Supplementary file 1 — Figure S1 – Improvement in number of peptide-spectrum matches from GOAT gradient optimization correlates inversely with the speed of the instrument used to acquire the data. [file pmic0014-1467-sd1.docx]

SUPPLEMENTARY MATERIAL FOR

**GOAT – A Simple LC-MS/MS Gradient Optimization Tool**

David C. Trudgian^1#^, Roman Fischer^2#^, Xiaofeng Guo^1^, Benedikt M. Kessler^2^ and Hamid Mirzaei^1*^

**Supplementary Methods**

***Sample Preparation & Mass Spectrometry – Oxford***

Jurkat cells were lysed using RIPA buffer and proteins precipitated with Chloroform and Methanol [[1](#_ENREF_1)] before resuspension in 6M Urea and 1/6 dilution with 100mM TEAB and addition of trypsin (Promega) in a 1/50 protein/enzyme ratio followed by digestion over night at 37 `C. Peptides were acidified (0.1% TFA final concentration), desalted (SepPak) and dried in a SpeedVac concentrator. For high pH fractionation Peptides were then resuspended in 20 mM ammonium formate (pH 10) and loaded onto a C18 cartridge (SOLA, Thermo Fisher). The Flow through (FT) was collected. Peptides were further fractionated by stepwise elution with increasing percentages of Acetonitrile (10%, 20% and 50%) in 20mM ammonium formate at pH 10. The four fractions were dried down and resuspended in loading buffer (1% Acetonitrile, 0.1% TFA).

On the Orbitrap Velos fractionated samples were analysed with a standard linear gradient of 3-40% in 60 or 120 minutes delieverd by a nanoAquity UPLC (Waters, column - 1.7um BEH130, C18, 75um x 250mm). Buffer A was 0.1% formic acid in water, buffer B 0.1% formic acid in Acetonitrile. A Top 20 CID method was used on the Orbitrap Velos. Resolution was set to 60000 (@400 M/z) in an M/z range of 300-2000. Isolation window was set to 1.5 Da and normalized collision energy was 35%. Selected precursors (Threshold 500 counts) were excluded for 15s.

Sample analysis on the Q-Exactive was conducted with a resolution of 70000 (@200 M/z) and a Top 15 method. Precursors with an M/z between 380 and 1800 were selected for MSMS with an isolation width of 1.6 Da and 28% normalized collision energy. Selected precursors (Threshold 10000 counts) were excluded for 27s. Standard gradient peptide separation was archived on a Dionex Ultimate 3000 UPLC (Thermo) and a nEasy column (2um Pepmap, C18, 75um x 500mm) using a linear gradient from 2-40% of buffer B (composition as above) in 57 minutes.

The GOAT optimized gradients for each sample are given in supplementary tables S11-15. All raw data is available for download – see Data Availability section below.

***Sample Preparation & Mass Spectrometry – UTSW***

*HeLa* cells were lysed in modified RIPA lysis buffer (50 mM Tris-HCl pH 7.4, 150 mM NaCl, 1% NP-40, 0.25% Na deoxycholate, 1 mM EDTA) containing a cOmplete Protease Inhibitor Cocktail (Roche Diagnostics, Laval, QC). After precipitation using trichloroacetic acid proteins were resuspended in digestion buffer containing 100 mM Tris pH 8.0, 50% trifluoroethanol, 6M urea, 2M thiourea, and 0.5% SDS. Reduction with 8mM Tris(2-carboxyethyl)phosphine (TCEP) at 37°C for 30 min was followed by alkylation with 12mM iodoacetamide (IAA) at 37°C for 30 min in the dark. Digestion was performed overnight at 37C with trypsin (Promega).

Strong anion-exchange fractionation (SAX) used a modified StageTip-based fractionation protocol [[2](#_ENREF_2)]. Peptides from 100ug protein were loaded on 200μl tip columns packed with 6 layers of 3M Empore anion-exchange disk (1214-5012, Varian, Palo Alto, CA). Britton & Robinson buffers (containing 20mM acetic acid, 20mM phosphoric acid, and 20mM boric acid) at pH 11.0, 8.0, 6.0, 5.0, 4.0, 3.0, and 2.0 were used for loading and elution. Digests were loaded in pH 11.0 buffer, and the flow-through and seven other eluates using pH 8.0, 6.0, 5.0, 4.0, 3.0, 2.0, and 80% acetonitrile with 0.1% TFA were collected sequentially.

Samples were analyzed using an Orbitrap Elite mass spectrometer (Thermo, Bremen) coupled to an Ultimate 3000 RSLC-nano HPLC system (Dionex, Santa Clara). Peptides were loaded onto a 75 µm i.d. x 50 cm column packed in-house with a reverse-phase material ReproSil-Pur C18-AQ, 1.9µm resin (Dr. Maisch GmbH, Ammerbuch-Entringen, Germany). Buffer A consisted of 2% ACN and 0.1% formic acid in water. Buffer B consisted of 80% ACN, 10% TFE and 0.08% formic acid in water. The analytical gradient was 0-28% buffer B in 100 minutes, followed by a ramp to 90% B in 5 minutes. The Orbitrap Elite acquired full-MS scans in the Orbitrap at 240,000 resolution. Data dependent analysis (DDA) with CID fragmentation was used. Up to 13 rapid-scan MS/MS spectra per cycle were acquired in the ion-trap.

The GOAT optimized gradients for each sample are given in supplementary tables S11-15. All raw data is available for download – see Data Availability section below.

***Data Analysis***

Raw mass-spectrometry data files were converted to Mascot generic format (MGF) using Proteowizard msconvert (version 3.0.3535) [[3](#_ENREF_3)]. Peptide and protein identification were performed using Oxford and UTSW installations of the Central Proteomics Facilities Pipeline (CPFP) version 2.0.3 [[4](#_ENREF_4), [5](#_ENREF_5)]. Database search was performed using X!Tandem (Tornado 2008.12.10.1) [[6](#_ENREF_6)] and OMSSA (2.1.8) [[7](#_ENREF_7)] against the UniProtKB whole proteome sequence database (release 2012_04) [[8](#_ENREF_8)]. For Orbitrap Velos and Elite data precursor and fragment tolerances were 20ppm and 0.5Da respectively. For QExactive data fragment tolerance was 0.1Da. All searches allowed 3 missed cleavages, and allowed peptide masses from the monoisotopic or first two 13C peaks. Concatenated target-decoy sequences were used to filter results to a 1% FDR at the peptide spectrum match (PSM) and protein levels.

***Preliminary Gradient Optimiziation Experiment***

Prior to the implementation of the GOAT method in software a preliminary study examined the effect of gradient optimization on whole-cell lysate. A whole cell lysate sample was run using a 1h standard analytical gradient (described above) on the Oxford Orbitrap Velos instrument. Following the basic method described in the main text an optimized gradient was generated manually using a spreadsheet. Reanalysis of the sample using this optimized gradient delivered an improvement in peptide spectrum matches from 8866 to 9306, and protein group identifications from 957 to 1037.

**RAW Data Availability – Chorus**

Raw MS data is available via the Chorus Project at <http://chorusproject.org>

Each analysis is available as a separate experiment within the project named ‘GOAT - Gradient Optimization’.

Anonymous download links are listed below:

<https://chorusproject.org/anonymous/download/experiment/-7438231320068507808>

<https://chorusproject.org/anonymous/download/experiment/4a09a321c4754d84b7469058564c6b32>

<https://chorusproject.org/anonymous/download/experiment/8439077896245945425>

<https://chorusproject.org/anonymous/download/experiment/5861461178024902920>

<https://chorusproject.org/anonymous/download/experiment/5209229584571243268>

<https://chorusproject.org/anonymous/download/experiment/5277524578419245898>

<https://chorusproject.org/anonymous/download/experiment/-410013579944669845>

<https://chorusproject.org/anonymous/download/experiment/-5342107700840477852>

**REFERENCES**

[1] Wessel, D., Flugge, U. I., A method for the quantitative recovery of protein in dilute solution in the presence of detergents and lipids. *Analytical biochemistry* 1984, *138*, 141-143.

[2] Wisniewski, J. R., Zougman, A., Mann, M., Combination of FASP and StageTip-Based Fractionation Allows In-Depth Analysis of the Hippocampal Membrane Proteome. *Journal of proteome research* 2009, *8*, 5674-5678.

[3] Kessner, D., Chambers, M., Burke, R., Agusand, D., Mallick, P., ProteoWizard: open source software for rapid proteomics tools development. *Bioinformatics* 2008, *24*, 2534-2536.

[4] Trudgian, D. C., Mirzaei, H., Cloud CPFP: A Shotgun Proteomics Data Analysis Pipeline Using Cloud and High Performance Computing. *Journal of proteome research* 2012.

[5] Trudgian, D. C., Thomas, B., McGowan, S. J., Kessler, B. M.*, et al.*, CPFP: a central proteomics facilities pipeline. *Bioinformatics* 2010, *26*, 1131-1132.

[6] Craig, R., Beavis, R. C., TANDEM: matching proteins with tandem mass spectra. *Bioinformatics* 2004, *20*, 1466-1467.

[7] Geer, L. Y., Markey, S. P., Kowalak, J. A., Wagner, L.*, et al.*, Open mass spectrometry search algorithm. *Journal of proteome research* 2004, *3*, 958-964.

[8] Consortium, U., Reorganizing the protein space at the Universal Protein Resource (UniProt). *Nucleic acids research* 2012, *40*, D71-D75.

QExactive

Orbitrap Velos

Orbitrap Elite

**Figure S1 –** Improvement in number of peptide-spectrum matches from GOAT gradient optimization correlates inversely with the speed of the instrument used to acquire the data. In each case methods used on the instruments in each laboratory were optimized for peak identification rates in protein identification submissions.
